# Advanced footwear technology in well-trained athletes: methodological insights from outdoor running

**DOI:** 10.3389/fphys.2025.1713902

**Published:** 2025-12-02

**Authors:** Borja Muniz-Pardos, Konstantinos Angeloudis, Irina Zelenkova, Fergus M. Guppy, Luis A. Marco-Contreras, Alejandro Gomez-Bruton, Gabriel Lozano-Berges, Yannis Pitsiladis, Jose A. Casajus

**Affiliations:** 1 EXER-GENUD (Growth, Exercise, NUtrition and Development) research group (S72_23R), FIMS Collaborating Center of Sports Medicine, University of Zaragoza, Zaragoza, Spain; 2 Faculty of Health and Sport Science (FCSD), Department of Physiatry and Nursing, University of Zaragoza, Huesca, Spain; 3 School of Energy, Geoscience, Infrastructure and Society, Institute of Life and Earth Sciences, Heriot-Watt University, Edinburgh, United Kingdom; 4 Faculty of Health Sciences, Universidad San Jorge, Villanueva de Gállego, Spain; 5 Department of Biology, Faculty of Science, Hong Kong Baptist University, Kowloon Tong, Hong Kong, China; 6 Department of Physiatry and Nursing, Faculty of Medicine, University of Zaragoza, Zaragoza, Spain

**Keywords:** variability, running economy, supershoes, records, methodological considerations, order effect

## Abstract

**Background:**

Advanced footwear technology (AFT) is reported to elicit an approximate 4% average improvement in running economy (RE). However, a large inter-individual variability remains unexplained, and limited research examined the impact of AFT during outdoor running. The aim was to compare the physiological, biomechanical and perceptual responses of 36 well-trained athletes to running outdoors using three different AFT and a traditional racing shoe.

**Methods:**

Thirty-six well-trained athletes (19 males and 17 females) had their maximal aerobic capacity ( $\dot{V}$ O_2_max) and anaerobic threshold (AT) determined in laboratory conditions and were familiarised to the different shoe running conditions. Within 7 days, athletes ran 4 × 6 min running bouts, paced outdoors at 95% of their individual AT with 10 min recovery, in three different AFT conditions and a traditional flat. Oxygen uptake ( $\dot{V}$ O_2_), heart rate, rating of perceived exertion (RPE), lactate, shoe perception, and biomechanical responses were compared between the four running trials.

**Results:**

No differences were observed in the RE between shoe conditions, with a great inter-individual variability (range: 12% impairment to 14% improvement in RE). This variability was accompanied by a significant $\dot{V}$ O_2_ order effect across exercise bouts (bout 2 lower than one [-1.1 mL/kg/min, *p* = 0.002]; bout 3 lower than 2 [-0.8 mL/kg/min, *p* = 0.027]; no differences between bouts 3 and 4). This variability was likely due to methodological issues such as one squared-wave RE measurement per shoe condition or the lack of a mirrored experimental design, among others. There was no order effect in other physiological or biomechanical variables. No significant differences were found in lactate, heart rate or rate of perceived exertion between running trials. Biomechanical responses to the different shoe conditions were also highly variable. One of the advanced AFT shoes showed a greater strike angle (+2.07°; *p* = 0.001), with no other significant differences between shoes conditions.

**Conclusion:**

The large variability in the physiological response to AFT may be explained by methodological considerations. A minimum of two-bout RE measurements, the use of a mirrored order, a sufficient familiarisation with shoes and experimental designs, among other considerations, seem crucial to enhance the ecological validity and reduce data variability.

## 1 Introduction

The impact of the introduction of advanced footwear technology (AFT) on running times has shown to be dramatic, as reflected by the plethora of–World, Olympic, European, National–records since 2016, and by recent observational studies (Bermon, 2021; Bermon et al., 2021; Senefeld et al., 2021; Goss et al., 2022). For example, Bermon et al. made reference to performance improvements ranging from 1.7% to 2.3% in female, and 0.6%–1.5% in male elite distance runners, respectively, when comparing their 2016 and 2019 season best times (Bermon et al., 2021). Similarly, Senefeld et al. (2021) examined the marathon finishing times of the top 50 elite male and female athletes at four World Marathon Major series in the 2010s before and after the introduction of AFT (restricted to Nike shoes or prototypes). Finishing times were 2.0% faster in male, and 2.6% faster in female athletes wearing AFT when compared to athletes wearing traditional shoes. This magnitude of performance advantage during a marathon race can be decisive. For example, during the women’s Tokyo 2020 Olympic marathon, the gold medal was won by a Kenyan athlete in a time of 02:27:20 h:min:s, while the athlete finishing the race in seventh place finished in a time of 02:29:36 h:min:s; a performance difference of 1.5%.

Different explanations have been suggested to justify the fast marathons observed in recent years. Joyner et al. proposed a multitude of factors including training advances, the use of AFT, the optimization of carbohydrate intake, drafting, pacing and novel ways of doping (Joyner et al., 2020). In contrast, our group and others, attribute the recent fast marathons to primarily technological advances, and not to other physiological, tactical or nutritional factors (Muniz-Pardos et al., 2020; Muniz-Pardos et al., 2021). Specifically, among the different factors proposed by Joyner et al., it can primarily be the introduction of AFT that could potentially explain such abrupt changes in performances since 2016 in road running (Bermon et al., 2021), and since 2020 in track running with the introduction of the so-called *superspikes* (Mason et al., 2023; Willwacher et al., 2024). As we discussed recently in a Viewpoint, no other factors impacting performance besides the evolution of technology, including novel shoe designs, have experienced such a transformation in recent years (Muniz-Pardos et al., 2024).

A defining study conducted by Hoogkamer et al. (2018), compared the energy cost of running in AFT and traditional racing shoes and reported a 4% average improvement in running economy (RE) and estimated that this energetic savings translated to a 3.4% improvement in running velocity at marathon world record pace (i.e., 20.6 km/h). Rodrigo-Carranza et al. examined in a systematic review and meta-analysis the pooled effect that AFT has on RE (n = 10 studies) and foot mechanics (n = 7 studies) (Rodrigo-Carranza et al., 2021). This meta-analysis reported that AFT with increased longitudinal bending stiffness (LBS) through a curved plate improved RE by 2.2% on average, when compared to a traditional flat. Additionally, these authors reported that this RE benefit was accompanied by an increased stride length and a longer contact time when running in AFT shoes, when compared to a traditional flat. Previous research has suggested that this biomechanical alteration is not only due to the increased LBS caused by the carbon plate but may also be caused by other mechanical mechanisms like the “*teeter-totter effect*” (Nigg et al., 2021) or the “slingshot effect” (Ortega et al., 2021). Healey and Hoogkamer highlighted that the energy savings likely result from an interaction of the highly compliant and resilient midsole, shoe geometry, and other effects of the shoe design not related to the LBS (Healey and Hoogkamer, 2022). The meta-analysis performed by Rodrigo-Carranza et al. must however be interpreted with caution as studies were counted multiple times during the analysis, so that studies including more experimental conditions had a greater influence on the pooled effect, which may be a source of bias.

Despite these remarkable early findings and predictions, the findings of later studies revealed different magnitude of effects and a greater variation in the response to AFT, with some studies showing changes in RE ranging from a 2%–6% benefit (Hoogkamer et al., 2018), other studies from a 0% to a 6% benefit (Hunter et al., 2019), and other studies showing much larger variations (10% improvement to 13% impairment in RE) (Hébert-Losier et al., 2022). An additional limitation in AFT research is related to the potential performance-enhancing effects of AFT in the female elite athlete population, which is much less studied (Langley et al., 2023). However, recent improvements in elite running performances across all distances appear to be more pronounced in women’s events, when compared to men (Mason et al., 2024). Mason and colleagues attributed this potential advantage to reduced body mass, smaller shoes, greater relative increases in leg length, greater stride frequency, and different muscle-tendon unit properties (Mason et al., 2024). Further research focused on potential sex-based differences would help understanding the impact AFT, especially in an ecologically valid environment (i.e., outdoors).

In addition to the large variability observed in previous laboratory studies and the intrinsic difficulty in comparing studies employing different AFT designs with different running flats, recent research highlighted the limitation to test AFT in the laboratory, especially as the use of bouncy treadmills may not reflect the real impact that AFT may have on the ground (Kram, 2022), with the treadmill absorbing energy from every step that could potentially be used by the shoe structure to return more energy to the runner. Nevertheless, no previous research has studied the impact of AFT in the RE of athletes exercising in outdoor conditions.

Therefore, the aim of our study was to compare the physiological, biomechanical and perceptual responses of well-trained male and female athletes to running outdoors using three different AFT shoes *versus* a traditional racing shoe.

## 2 Methods

### 2.1 Study population

Forty well-trained distance runners/triathletes (21 females and 19 males) were initially recruited, representing one of the largest AFT studies to date involving well-trained athletes. The inclusion criteria for athlete participation in the present study were to be well-trained, healthy, and with personal bests sub 35:00 min:s in 10 km or 17:00 min:s in 5 km in males, or sub 40:00 min:s or 19:00 min:s, respectively, in females. Upon recruitment, all subjects completed and signed an informed consent to participate in the study. The present study followed the Declaration of Helsinki 1961 (revision of Fortaleza 2013) and was approved by the Ethics Committee of Aragon (CEICA) with the registration number PI22/305.

### 2.2 Experimental design

The present study design required runners to attend the University of Zaragoza on two separate occasions separated by at least 3 days (median of 7 days [3–11 days]) to avoid any residual fatigue between visits. The first visit comprised a maximal oxygen uptake ( $\dot{V}$ O_2_max) and ventilatory threshold test in the laboratory, anthropometric assessment, and shoe familiarisation. The second visit involved assessing RE in response to four randomised shoe conditions outdoors. The RE trials were conducted on a 468 m cement lane surrounding a 400 m tartan track in order to reproduce an ecologically valid experiment.

### 2.3 Shoe conditions

Three different models of AFT shoes were tested and were compared to a traditional racing shoe (main characteristics detailed in Table 1). AFT 1 was claimed to be the best road running shoe of a very popular brand by the time of these experiments, while AFT 2 was the improved version of AFT 1. Finally, AFT 3 was the best road running shoe of the main competitor. All the shoes were labelled so each athlete used the same shoes during both familiarisation and RE trials (each athlete had their own shoes, for each shoe condition).

**TABLE 1:** Shoe characteristics for the control shoe and the three AFT shoes (for a US 9 size).

Shoe characteristics	Control shoe	AFT 1	AFT 2	AFT 3
Weight (grams)	229	212	215	185
Heel stack height (mm)	27	39	39.5	39
Metatarsal area stack height (mm)	19	30.5	33	31
Heel-to-toe offset (mm)	8	8.5	6.5	8
Midsole foam material	EVA	TPE + EVA	TPE + EVA	Pebax
Carbon fiber system	-	Carbon rods	Carbon rods	Full plate

AFT, advanced footwear technology; EVA, etilvinikacetato; Pebax, Poliether block Amida; TPE, thermoplastic elastomer.

### 2.4 Visit 1. Maximal oxygen uptake and ventilatory threshold determination

During the first laboratory visit, informed consent was obtained followed by a medical history and pre-participation screening. Subjects lay supine for 5 min for a resting electrocardiogram and blood pressure. Anthropometric assessment included body mass (using bioimpedance; TANITA BC 780-S MA, Tanita Corp., Tokyo, Japan), height (stadiometer SECA 225, SECA, Hamburg, Germany; to the nearest 0.1 cm), height from sitting position, and foot length.

Maximal aerobic capacity test. All subjects had previous experience with $\dot{V}$ O_2_max testing. Following a self-paced warm-up on a treadmill (h/p/cosmos, Nussdorf–Traunstein, Germany), subjects were instrumented with a portable metabolic analyzer (Cosmed K5, Cosmed Srl, Rome, Italy) and a heart rate device (Polar H10, Polar Electro, Kempele, Finland) prior to commencing the test. The $\dot{V}$ O_2_max protocol consisted of a 3-min run at 10 km/h on a 1% gradient, followed by increases of 1 km/h/min until volitional exhaustion. Mask size was individualised to be fitted for each individual during the first visit, and the same size was used for visit 2. Heart rate was monitored throughout the test, and overall perception of effort (RPE) and specific RPE for the legs were registered immediately after the test. This test aimed to determine $\dot{V}$ O_2_max (defined as the highest 20-s mean values obtained during the test) and the individual anaerobic threshold (AT), determined through visual assessment by two experienced exercise physiologists. The references for the AT determination were the detection of the second increase of the $\dot{V}$ CO_2_ dynamics, the exponential increase in the ventilation, an increase of both the $\dot{V}$ O_2_ (VE/ $\dot{V}$ O_2_) and VCO_2_ (VE/ $\dot{V}$ CO_2_) equivalents, or through a decrease in the end-tidal CO_2_ pressure. Individual speeds for subsequent shoe trials were determined at the 95% of the AT velocity. During the $\dot{V}$ O_2_max test, athletes wore their preferred non-technology shoe. This test served to objectively quantify the individualized running speed for the RE trials. An intensity near to but slightly under the AT was selected given that the running shoes tested were designed for running at high velocities but at the same time avoiding the slow component of oxygen uptake during the RE trials. Visit one also involved a familiarisation of the different running shoes during a light 3–5-min run with each pair of shoes in preparation for visit two and serving as cool down after the $\dot{V}$ O_2_max test.

### 2.5 Visit 2. Running economy test

**FIGURE 1:**
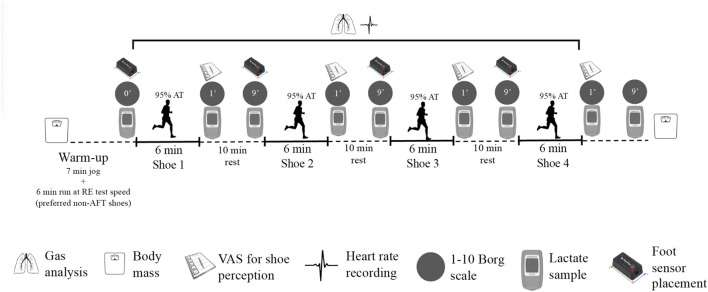
Scheme of the measurements during the running economy tests. AFT, advanced footwear technology; AT, anaerobic threshold; RE, running economy; VAS, visual analogue scale.

During the second visit conducted 7 [3 to 11] days later, indices of performance with particular focus on RE, physiological and perceptual response, and foot mechanics were assessed for each shoe condition determined on the 468-m cement lane surrounding the 400 m University of Zaragoza track (Schematic displayed in Figure 1). Air Temperature and humidity were recorded at the beginning and the end of the experimental sessions. In case of a change of weather during the test (rain or hard wind waves), tests were suspended and postponed. Body mass was measured before and after each RE trial. The order of shoe conditions was randomly assigned for each athlete using the formula = RANDBETWEEN (1; 4) in Excel. Runners were required to avoid intense exercise for at least 24 h prior to the test, caffeine and food ingestion during the last 3 h prior to the test. Each runner warmed up for 7 min (self-paced but slower than the running speed of the RE test) in their own choice of training shoes, prior to being instrumented with the same portable metabolic analyser used during Visit 1. Prior to each full testing session (i.e., for each participant), the K5 metabolic analyser was warmed up for a minimum of 30 min and calibrated following the manufacturer instructions. This consisted of 1) a room air calibration, 2) a flow meter calibration using a 3-L calibration syringe, 3) a scrubber calibration that zeros the CO_2_ analyzer, 4) reference gas calibration (16% O2, 5% O2, 79% nitrogen), and 5) a delay calibration for the BxB mode.

Thereafter and serving as a specific warm up for a further familiarisation with the protocol, athletes performed two further laps (total of 936 m) at their individualised RE test pace with their own shoes and instrumented with the K5 analyser, following a 10 min rest before the first bout. Pre-trial blood lactate was collected from a single drop of whole blood from the fingertip using a lactate meter (Lactate Pro 2, Arkray Europe, B.V., Amstelveen, the Netherlands), and pre-trial heart rate and RPE were also collected. Each athlete performed four 6-min exercise bouts at 95% of their AT velocity (one per shoe condition, with shoes in randomized order), with 10-min rest in between. Heart rate and ventilatory parameters were monitored throughout the RE trial, while blood lactate, whole-body RPE and legs-only RPE collected at min one and nine of recovery post each shoe trial. A researcher paced all runners at their individual speed using a bicycle. Four cones were placed every 117 m so that the pace was carefully controlled by the pacer and by two other researchers with a timer. The RE elicited by each shoe condition was determined as the mean $\dot{V}$ O_2_ during 3–5 min to ensure steady state. To reduce the noise in the ventilatory measurements, any measurements more than two standard deviations from the mean of the 7-breaths periods were removed, and the 7-breath average method subsequently applied. A 10-point visual analogue scale for stability (from 0 = very unstable to 10 = very stable), cushioning (from 0 = very stiff to 10 = very soft), energy return (from 0 = very low to 10 = very high), forward propulsion (from 0 = no propulsion to 10 = a lot of propulsion), comfort (from 0 = very uncomfortable to 10 = very comfortable), weight (from 0 = very heavy to 10 = very light), and overall performance (from 0 = very bad to 10 = very good) were completed by all runners immediately after each AFT condition.

Foot mechanics were assessed during each shoe trial using wireless inertial measurement units (IMUs; Physilog5, GaitUp, CH, Lausanne, Switzerland; weight: 19 g, size: 50 × 37 × 9.2 mm). An IMU was placed on the external side of both the right and left foot before each 6-min bout, and measured both 3D acceleration and 3D angular velocity at 256 Hz. The accelerometer range was set to ±16 g and ±2000°/s for the gyroscope and 64 Hz for the barometer. Prior to each running trial, a functional calibration method [for full details see (Falbriard et al., 2018)] was used to automatically align the technical frame of the IMUs with the functional frame of the foot, and this required the athletes to maintain a static standing position of 30 s prior to each RE test. Contact time (ms), cadence (steps/min), strike angles (^o^), stride time (s), stride length (m), strike eversion (^o^), horizontal impact (g; impact accelerations experienced by the runners in the X-axis), and vertical impact (g; impact accelerations experienced by the runners in the Y-axis) were assessed during all trials, and these data were averaged from min three to min five of each running bout to match with the ventilatory data. A summary of the second visit is presented in Figure 1.

### 2.6 Statistical analysis

All variables included in the statistical analysis underwent the Shapiro-Wilk normality test. For normally distributed variables, one-way repeated measures ANOVA was used to identify any potential effect of running shoes on RE, foot mechanics, perception and lactate responses. When there was a significant effect of the shoe condition, *post hoc* Bonferroni correction for multiple comparisons was applied to examine potential differences between shoe conditions. Greenhouse-Geisser correction was used when Mauchly’s test of sphericity was significant. For variables that were not normally distributed, Friedman’s repeated measures ANOVA was used and, when significant interactions were found, Durbin-Conover pair-wise comparisons were reported. Statistical analysis was performed with Jamovi (v 2.3.18).

## 3 Results

### 3.1 Subjects

A final sample of 36 athletes (19 males and 17 females; Table 2) completed the present study. Four participants withdrew due to the following reasons: lack of interest (n = 1), the protocol interfering with competition schedules (n = 1), unavailability of shoes size (n = 1), and inability to perform the RE test at the required speed (n = 1). The included athletes were national to international level triathletes (n = 7), middle-distance runners (n = 15) and long-distance runners (n = 14).

**TABLE 2:** Descriptive characteristics of participants included in the study.

Personal and physiological characteristics	Males (n = 19) Mean [SD]	Females (n = 17) Mean [SD]
Age (y)	25.6 [6.4]	24.3 [7.6]
Weight (kg)	66.3 [5.0]	52.8 [3.0]
Height (cm)	179.5 [5.3]	164.2 [3.4]
SB 10 k (min:s)	30:35 [1:30] (n = 14)	37:11 [01:42] (n = 10)
SB 3000 m (min:s)	08:35 [00:22] (n = 5)	
SB 1500 m (mins:s)		04:43 [00:16] (n = 7)
Tr volume (km/week)	90 [35]	74 [24]
$\dot{V}$ O_2_ max (mL/kg/min)	73.4 [5.4]	62.0 [4.6]
HR max (bpm)	186.3 [10.7]	180.2 [11.2]
AT speed (km/h)	17.3 [1.3]	14.7 [0.9]
% $\dot{V}$ O_2_ max at AT	78.5 [4.4]%	83.4 [3.0]%
95% AT speed (km/h)	16.3 [1.0]	14.0 [0.8]

HR, heart rate; AT, anaerobic threshold; SD, standard deviation; SB, season best; Tr, training; $\dot{V}$ O_2_ max, maximal oxygen uptake.

### 3.2 Running economy testing

**FIGURE 2:**
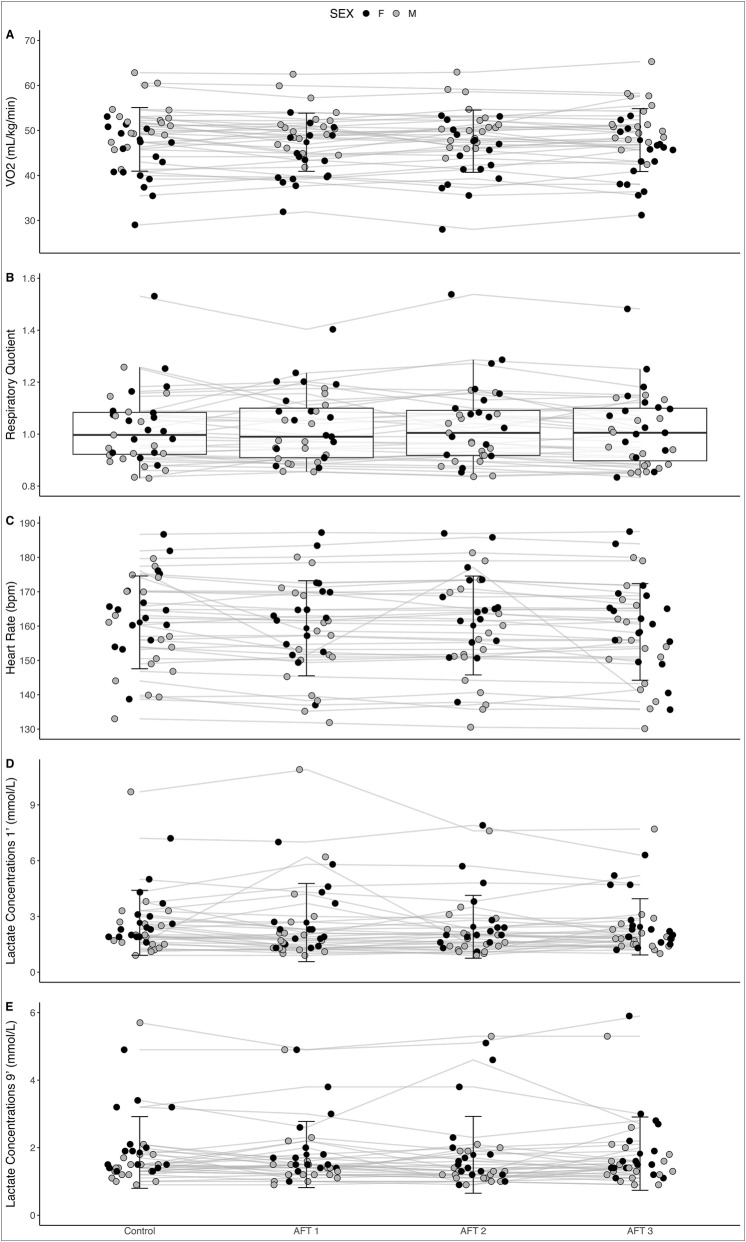
Running economy **(A)**, respiratory quotient **(B)**, heart rate **(C)**, and lactate 1 min **(D)** and 9 min **(E)** post exercise when running in four different shoe conditions in well-trained athletes (n = 36). Mean and standard deviation are presented except for respiratory quotient given its not normal distribution, showing median and confidence intervals.

Ambient temperature before and after the running economy test was not different (15.5 ± 4.0 and 15.4 ± 3.8 °C, respectively; *p* = 0.819), while relative humidity was significantly reduced after the test, when compared to pre-test values (44.3% ± 8.6% and 47.1% ± 10.1%, respectively; *p* = 0.046). Running economy and metabolic cost values for each shoe are shown in Table 3. There was no main effect of AFT on RE when examining the whole sample (F = 0.99, η^2^_p_ = 0.03, *p* = 0.400). Similar results were observed in male and female subjects when analysed separately. No significant interaction between AFT and sex was observed (F = 0.98, η^2^_p_ = 0.03, *p* = 0.405; Figure 2). When exploring further sub-group analyses according to the athletes’ profiles (middle/long distance runners), no significant interaction between AFT and athlete profile was observed (F = 0.44, η^2^_p_ = 0.01, *p* = 0.723).

**TABLE 3:** Oxygen consumption ( $\dot{V}$ O_2_), energy cost of transport (eCOT) and metabolic cost during the running economy test for each shoe condition.

Running economy and metabolic cost variables	CON Mean [SD]	AFT 1 Mean [SD]	AFT 2 Mean [SD]	AFT 3 Mean [SD]
Males (n = 19)				
$\dot{V}$ O2 (mL/kg/min)	51.8 [5.3]	50.6 [5.2]	51.0 [5.0]	51.5 [5.4]
eCOT (mL/kg/km)	196.6 [20.2]	191.7 [15.2]	193.5 [18.2]	195.6 [18.4]
Metabolic cost (W/kg)	18.3 [1.7]	17.9 [1.7]	18.0 [1.6]	18.2 [1.8]
Females (n = 17)				
$\dot{V}$ O2 (mL/kg/min)	43.8 [6.5]	43.8 [6.0]	43.9 [7.0]	43.7 [6.3]
eCOT (mL/kg/km)	191.2 [27.9]	191.1 [25.3]	191.2 [29.0]	190.5 [25.0]
Metabolic cost (W/kg)	15.8 [2.3]	15.8 [2.1]	15.8 [2.4]	15.7 [2.2]

**FIGURE 3:**
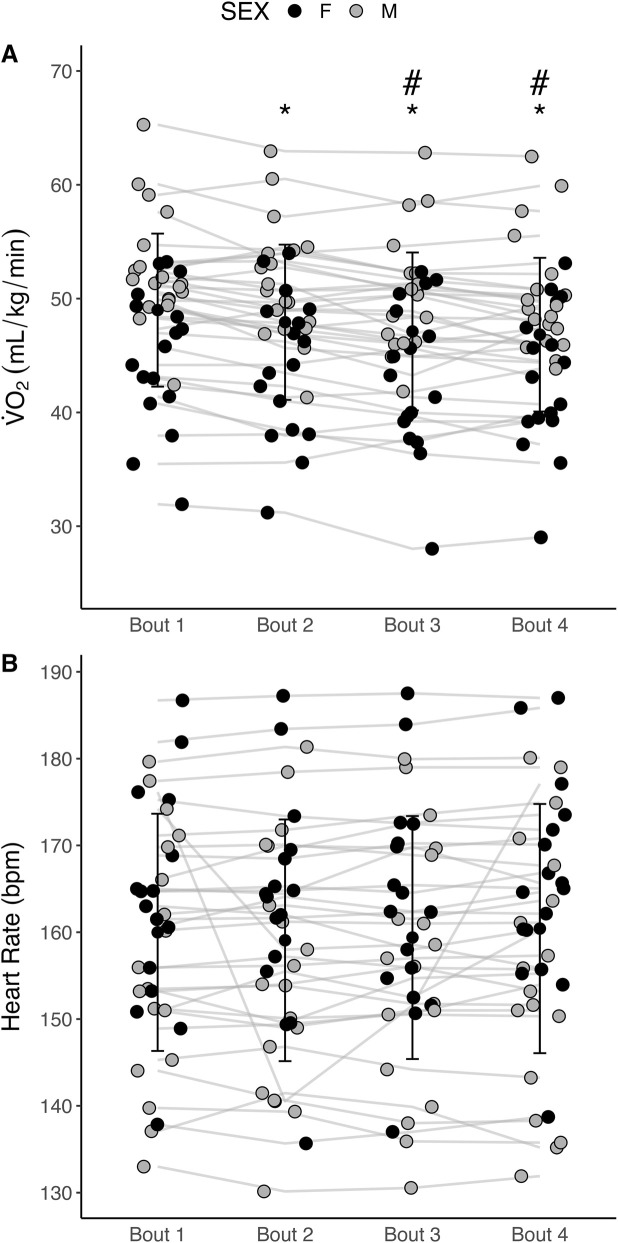
Oxygen uptake **(A)** and heart rate **(B)** across the four exercise bouts regardless of the shoe condition used (n = 36). *Significantly different to Bout 1; #Significantly different to Bout 2.

However, and most importantly, the findings of the present study are biased by an order effect in the $\dot{V}$ O_2_ variable as reflected by the ANOVA analysis, which revealed a significant within-subject effect of the bouts order, regardless of the shoe used (F = 17.5, η^2^_p_ = 0.333, *p* < 0.001; Figure 3). Further Bonferroni *post hoc* comparisons showed that the oxygen uptake of bout two was significantly lower than bout 1 (mean difference = 1.1 mL/kg/min, t = 3.9, *p* = 0.002); bout three was significantly lower than bout 2 (mean difference = 0.8 mL/kg/min, t = 3.0, *p* = 0.027) and bout 1 (mean difference = 1.9 mL/kg/min, t = 5.2, *p* < 0.001); and bout four was significantly lower than bout 2 (mean difference = 1.1 mL/kg/min, t = 3.4, *p* = 0.011) and bout 1 (mean difference = 2.2 mL/kg/min, t = 5.1, *p* < 0.001). No differences in the oxygen uptake were observed between bouts three and 4 (t = 1.0, *p* = 1.000).

### 3.3 Heart rate, lactate concentrations and RPE

Heart rate, lactate and RPE did not show any order effect (Figure 3). Heart rate was collected for 34 athletes (18 males and 16 females) while lactate concentrations were obtained for 33 athletes (18 males and 15 females). In male athletes, there was a significant interaction of heart rate (F = 3.82, *p* = 0.015). However, Post Hoc comparisons did not reveal any significant differences between shoe conditions. In female athletes, there was no significant effect of heart rate (F = 2.16, p = 0.106). In males, lactate as measured 1 min (χ^2^ = 3.5, *p* = 0.319; Figure 3) and 9 min after the run (χ^2^ = 0.7, *p* = 0.870; Figure 3) were not significantly different between AFT conditions. Similarly, in females, lactate levels as measured 1 min and 9 min after the run were not significantly different between AFT conditions (χ^2^ = 2.9, *p* = 0.404; Figure 3 and χ^2^ = 1.9, *p* = 0.588; Figure 3, respectively for min one and 9). No differences in RPE measured 1 min after the run (χ^2^ = 15.3, p = 0.810) and 9 min after the run (χ^2^ = 15.7, *p* = 0.787) were observed. Similarly, no differences were found in legs-only RPE 1 min after the run (χ^2^ = 24.9, p = 0.253) and 9 min after the run (χ^2^ = 10.6, p = 0.909).

### 3.4 Biomechanical response to AFT

**FIGURE 4:**
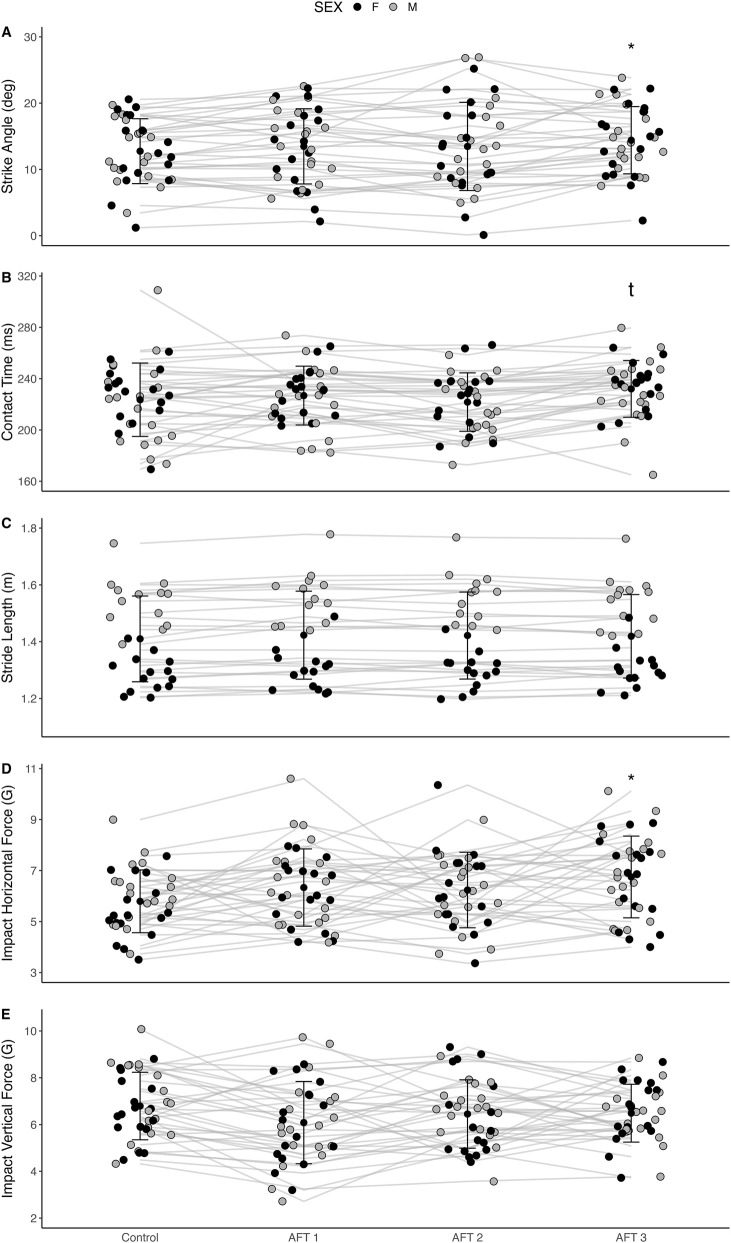
Strike angle **(A)**, contact time **(B)**, stride length **(C)**, impact horizontal force **(D)** and impact vertical force **(E)** as assessed with the Inertial Measurement Unit (IMU; n = 32). *refers to significant difference when compared to control shoe; t refers to a trend of p = 0.085 when compared to control.

Biomechanical variables were obtained from 32 athletes (18 males and 14 females), with four omissions due to technical problems with the sensor placement or data collection. In male athletes, there was a statistical trend on the strike angle (F = 3.31; *p* = 0.051; Figure 4) between shoe conditions. Post Hoc comparisons revealed a significantly greater strike angle in the AFT 3 when compared to the control shoe (mean difference = 2.07°; *p* = 0.001), but not to other AFT shoes. Additionally, there was a trend in the interaction of stride length (F = 2.84; *p* = 0.051; Figure 4), with no significant differences between shoe conditions from the *post hoc* comparisons. There was however no significant interaction of contact time (F = 2.57; *p* = 0.102), cadence (F = 1.23; *p* = 0.309), stride time (F = 1.5; *p* = 0.227), vertical impact (F = 2.33; p = 0.090) or horizontal impact (F = 1.24; *p* = 0.304) in male athletes. In female athletes, contact time (F = 3.09; p = 0.038; Figure 4) was significantly different between shoe conditions, with AFT 3 showing a trend towards a greater contact time when compared to control shoe (mean difference = 5.72 m; *p* = 0.085). Horizontal impact also showed to be different between shoe conditions (F = 2.84; *p* = 0.051; Figure 4), with the AFT 3 shoe showing a trend towards a greater horizontal impact than the control shoe. However, cadence (F = 1.16; *p* = 0.339), stride length (F = 1.56; *p* = 0.213), stride time (F = 1.36; *p* = 0.269), strike angle (F = 0.94; *p* = 0.388) and vertical impact (F = 0.48; *p* = 0.700) were not different between shoe conditions.

### 3.5 Shoe perception

**FIGURE 5:**
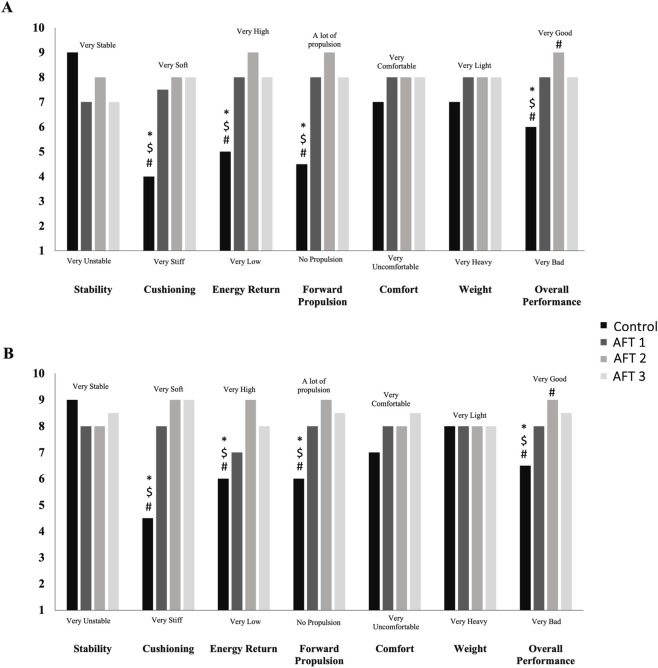
Median values from the visual analogue scales (1–10) in stability, cushioning, energy return, forward propulsion, comfort, weight, and overall performance for all four shoe conditions in males **(A)** and females **(B)**. #Significant differences to AFT 1; $Significant differences to AFT 2; *Significant differences to AFT 3.

The results from the shoe perception VAS are summarized in Figure 5. Both male and female athletes (in the whole sample) perceived a significantly greater *Cushioning* (Males: χ^2^ = 30.5, p < 0.001; Females: χ^2^ = 30.6, p < 0.001), *Energy return* (Males: χ^2^ = 27.1, p < 0.001; Females: χ^2^ = 28.0, p < 0.001), *Forward propulsion* (Males: χ^2^ = 34.6, p < 0.001; Females: χ^2^ = 29.2, p < 0.001) and *Comfort* (Males: χ^2^ = , p; Females: χ^2^ = 8.2, p = 0.042) when running in any of the AFT shoes, when compared to Control (Figure 5). No differences were found between shoe conditions in *Stability* or *Weight*. Additionally, male athletes reported to feel a significantly greater energy return when wearing the AFT 2 shoe than AFT 1 (Durbin-Conover statistic = 2.57, p = 0.014) and AFT 3 (Durbin-Conover statistic = 3.46, p = 0.001) (Figure 5). When rating the overall performance of each shoe, all AFT shoes were rated significantly higher than Control (Males: χ^2^ = 27.1, p < 0.001; Females: χ^2^ = 25.1, p < 0.001). Additionally, AFT 2 was rated significantly greater than AFT 1 shoe in both males and females (males: Durbin-Conover statistic = 2.22, p = 0.032; females: Durbin-Conover statistic = 2.30, p = 0.026) and AFT 3 only in males (Durbin-Conover statistic = 2.69, p = 0.010) (Figure 5).

## 4 Discussion

To our knowledge, this is the first study to examine the effect of AFT shoes on the RE of well-trained male and female athletes exercising outdoors. When looking at the differences in RE between shoe conditions without considering the relevant order effect identified in our study, we would interpret that AFT had no effect on our sample of well-trained athletes, with some athletes improving their RE by approximately 14%, while others experiencing a 12% RE impairment when compared to the control shoe. If ignoring the significant order effect and the important limitation of designing a single exercise bout per shoe condition, these findings would be in agreement with previous research showing great inter-individual variability between athletes (Hunter et al., 2019; Knopp et al., 2023), suggesting that some athletes may respond positively to this technology while others do not.

However, we identified an order effect in the main outcome variable ( $\dot{V}$ O_2_) across exercise bouts irrespective of the shoe used, which means that athletes were becoming more efficient across the exercise bouts (irrespective of the shoe used) until they reached the third bout, which was not different from the fourth bout. This is something we did not expect given that all athletes tested all the experimental shoes at the end of visit 1, and during visit 2 we implemented what we believed a sufficient warm up prior to the first exercise bout (8 min run +936 m run at the testing pace fully instrumented). However, it appears that this procedure may not be sufficient, especially if performing a single exercise bout per shoe condition. Considering this methodological issue, it is possible that previous research showing similar inter-individual variability to our study may have been exposed to the same limitation. For example, Hébert-Losier et al. (2022) examined the effects of running in AFT shoes on RE, showing a great inter-individual variability in RE ranging from – 10.3% to 13.3%. This research included an even shorter warm-up phase during the experimental trials (2-min warm-up at self-selected pace prior to the first bout) and a single trial per shoe, which would have likely elicited an insufficient warm-up and an aggravated variability biased by such methodological limitations. Day and Hahn reported in their study that seven out of nine runners were more economical with the control shoe than in the AFT shoe (Day and Hahn, 2019). While these authors used a 2-squared wave design, a total of 12 5-min running bouts (2 speeds x three shoe conditions x two trials per shoe) could have caused residual fatigue across exercise bouts, especially for the faster speeds (17 km/h). Additionally, no specific details about the warm-up were provided (self-selected warm-up).

Barrons and colleagues highlighted the need for multiple trials (at least two per shoe condition) to acquire reliable data when assessing RE in AFT studies given that metabolic analysers are prone to substantial measurement errors (Barrons et al., 2024; Van Hooren et al., 2024). This could however limit the number of shoe conditions tested within the same session to avoid an excessive number of running bouts. In addition to this suggestion by Barrons, a further way to reduce variability has been used by other authors, which would be to implement a mirrored order with two RE measurements for each shoe condition (for example, for four shoe conditions: ABCDDCBA) (Hébert-Losier et al., 2025; Perry et al., 2025). These two important strategies should be prioritised by future AFT research to allow for accurate data collection. There are other methodological limitations identified in the present research which are discussed below, and that could help further researchers to design rigorous AFT study designs.

In a recent study published by our group, Knopp et al. (2023) revealed a large variability in both world-class Kenyan road runners (RE change range: 11.4%–11.3%) and amateur Europeans (RE change range: 9.7%–1.1%) when running in different AFT shoes compared to a flat in a laboratory setting. In this case, a single bout per shoe and a 6-min warm-up were used, however, the authors did not find a $\dot{V}$ O_2_ order effect. In this case, the high variability was attributed to different reasons such as the use of different speeds across the subjects, different fitness levels, the use of different AFT (and control) shoes, variation in treadmill properties between different studies, or different foot mechanics patterns (Hébert-Losier et al., 2022). Further research considering the highlighted methodological limitations should be performed to elucidate the real impact of AFT on RE.

Besides the aforementioned limitations, an insufficient familiarisation with the exercise procedures and, especially, with AFT shoes may have contributed to such high variability (athletes may have been getting used to the procedures and shoes during the first bouts). Similar to our study, Hébert-Losier et al. (2022) included 3-min cool-down run at the end of the $\dot{V}$ O_2_max test with each of the two AFT shoes during visit 1 as a familiarisation session. This potentially insufficient shoe familiarisation in preparation for the RE session could have explained the large inter-individual variability found by the authors (range: 10.3%–13.3%). In our study, some athletes who were not accustomed to running in AFT shoes reported having ‘weak ankles’ after the end of visit 2. This may partly explain the variability also seen in the biomechanical variables (Figure 4). These limitations may justify the disparity between the shoe perception data (i.e., in some cases, the preferred shoe chosen by an athlete could be the least economical; Figure 5) and the physiological response to AFT. Another potential confounder of unknown significance, was the inability to blind the athletes to the different shoe conditions, and historical bias as many athletes had pre-conceived original preferences for one brand or another, which likely biased their response. Hébert-Losier et al. spray-painted the experimental shoes during their research (Hébert-Losier et al., 2022). However, a high variability was still witnessed. In a more recent study, Hébert-Losier et al. (2025) spray-painted one of the two same AFT shoes, but informed the runners that the painted shoe was the same but with the advanced technology removed (regular foam and no carbon fibre plate). Despite eliciting equal physiological and biomechanical effects, 87.5% of the runners preferred the non-painted shoe. This illustrates the great placebo effect on the athletes’ perception.

In addition to such potential sources of bias, the fact that some athletes were not experienced to running behind a bicycle or running while wearing a mask could have altered their RE during the first exercise bouts, with future studies ideally selecting more suitable pacing methods such as wave light pacing technologies (i.e., LED lights on the inside of athletics tracks), and including longer familiarisation trials. Additionally, the inclusion of middle-distance runners within our sample of athletes could also be a confounding factor as these athletes seems to be more economical at faster speeds (i.e., >19 km/h), but have a reduced performance at slower speeds, when compared to long-distance runners (Daniels and Daniels, 1992). Therefore, their physiological and biomechanical response to lower speeds could potentially elicit larger variability ranges. Future studies should ensure that experimental RE trials do not commence until an acceptable familiarisation is observed as evidenced by a <5% coefficient of variation in the main outcomes.

Other methodological consideration for future studies to reduce further variability in the response to AFT shoes is related to the selection of the control shoe. There is a wide variety of shoe models used in the literature as a control shoe [e.g., Nike Zoom Streak (Hoogkamer et al., 2018; Hunter et al., 2019), Adios BOOST one to three models (Hoogkamer et al., 2018; Barnes and Kilding, 2019; Hunter et al., 2019), athletes’ own shoe, Saucony Endorphine flat (Hébert-Losier et al., 2022), the Asics-Hyperspeed (Joubert and Jones, 2022) or the Nike Free 5.0 (Cigoja et al., 2020)]. This wide variability in the control shoe selection further difficult the comparison between studies. In our study we used a traditional racing flat as control shoe; a shoe that was widely used by elite runners prior to the introduction of AFT. The margin of improvement that AFT shoes may elicit in this case may be reduced when compared to previous studies using a lower quality control shoe.

With regard to the biomechanical response to the different AFT conditions, we also observed a high variability in these parameters as displayed in Figure 4. As the present study is the first to compare the kinematic response of different AFT shoes to a control shoe during outdoor running, these unique findings require further replication and should be cautiously compared with similar studies conducted indoors using a motorized treadmill. In our study, we observed a greater strike angle (i.e., adoption of a greater rearfoot strike pattern) in male runners when running on one of the AFT shoes (AFT 3), when compared to control. Additionally, the AFT 3 shoe elicited a greater horizontal impact and a longer contact time in females when compared to the control shoe. Our results differ from previous findings performed in the laboratory, such as the study of Hoogkamer et al. (2018), which found that AFT elicited longer contact times, reduced cadence and greater peak vertical ground reaction forces than the control shoe in male athletes. Nevertheless, 1 year later, the same authors were unable to replicate their own previous findings when conducting a more comprehensive biomechanical study (Hoogkamer et al., 2019). Hoogkamer et al. (2019) did not observe differences in contact time, but confirmed a greater peak vertical ground reaction force and reported a greater aerial time when running using AFT, when compared to the control shoe. Barnes and Kilding (Barnes and Kilding, 2019) found greater contact times in the control shoe when compared to AFT in a group of runners of a similar performance level to our sample, with no difference in cadence reported between shoes. These authors found slightly longer strides in men when running in AFT at lower speeds (14 km/h) but not at higher speeds (16 and 18 km/h), when compared to the control shoe. However, the opposite effect was observed in the female athletes in the study by Barnes and Kilding (Barnes and Kilding, 2019), with AFT eliciting 1% shorter strides when compared to the control shoe at lower speeds (14 km/h) but no differences at higher speeds (15 and 16 km/h). This study by Barnes and Kilding (Barnes and Kilding, 2019), reported an average RE benefit of 4.2% in the AFT shoe, when compared to the control shoe. However, the study failed to find consistent findings with regard to the biomechanical data collected. Barnes and Kilding also aimed to develop multiple linear regression models using the same biomechanical variables than in our study (i.e., contact time, stride length, flight time and cadence) to potentially predict RE changes. However, these variables only explained <1% of the average 4.2% RE savings, suggesting that there may be other biomechanical variables such as angular velocities of limb segments and joints, or ground reaction forces that are more reliable when aiming to explain RE variations. In other words, the large inter-individual variability in RE in response to AFT, not confined only to the present study remains unclear and may be explained by biomechanical features related to individual running style characteristics and not RE *per se*.

Taking the measures proposed to address the methodological issues raised in the present study will complicate further AFT studies involving elite athletes but are necessary to reduce variability and reduce the potential for both type I (false-positive) and type II (false-negative) errors. Studies such as the present investigation involving elite athletes during a competition period are particularly prone to such methodological issues that may have biased the real impact of AFT on RE and the comparison between shoe conditions.

The present study is the first to examine the effects of different AFT models outdoors, and revealed important methodological limitations needing consideration in future AFT research. For example, the need to extensively familiarise with the experimental protocol and shoe conditions. The selection of the speed for the RE tests should be further explored. In our study we selected a speed slightly under the anaerobic threshold to ensure metabolic stability and avoid the slow component of oxygen, but this may be an issue since AFT is designed to run fast (at or even above the anaerobic threshold). Finally, testing several shoe conditions entail long-lasting testing sessions which could cause significant changes in body mass throughout the test. This may have increased the RE variability throughout the test. Accounting for these changes, as well as for hydration status and fluid ingestion could help reducing the variability in long-lasting trials. In their recent study, Mazini et al. adjusted $\dot{V}$ O_2_ calculations for body mass changes during each test, which could be a solution for this (Zanini et al., 2025). Environmental conditions are also prone to change in such long-lasting tests performed outdoors. In our study, ambient temperature remained the same before and after the test, but humidity slightly changed (2.8%), which could potentially influence energy expenditure during exercise.

## 5 Conclusions and recommendations for future research

This is the first study to examine the acute effects of different models of AFT on physiological, biomechanical and perceptual responses during outdoor running in well-trained male and female runners. The large inter-individual variability observed between athletes may be explained by the methodological constraints detailed above, with particular importance of the use of a single trial per running shoe and the order of the shoe conditions, failing to employ a mirrored order. Future studies focused on the comparison of shoe conditions during outdoor running should (1) perform extensive familiarisation trials (with shoe conditions and also with the experimental design of the study), (2) examine different biomechanical features not studied in the present research (e.g., angular moments), (3) explore the long-term effects of running in AFT shoes and how non-familiarised athletes adapt to AFT shoes, (4) use more reliable pacing methods (e.g., wave light technologies rather than pacing with a cyclist), (5) ensure a good racing flat is used as a control condition, like the one used in our study, which would reveal the real benefit of AFT shoes, (6) recruit a homogeneous sample of athletes (e.g., marathon runners only), so their physiological response to the same fraction of AT is comparable, and most importantly, (7) use two or more square-wave RE tests for each shoe condition and a mirrored order as recently suggested (Barrons et al., 2024; Perry et al., 2025), so the energy expenditure for each experimental condition is verified and reliable.

## Data Availability

The raw data supporting the conclusions of this article will be made available by the authors, without undue reservation.
